# CSF level of β-amyloid peptide predicts mortality in Alzheimer’s disease

**DOI:** 10.1186/s13195-019-0481-4

**Published:** 2019-03-28

**Authors:** Adla Boumenir, Emmanuel Cognat, Severine Sabia, Claire Hourregue, Matthieu Lilamand, Aline Dugravot, Elodie Bouaziz-Amar, Jean-Louis Laplanche, Jacques Hugon, Archana Singh-Manoux, Claire Paquet, Julien Dumurgier

**Affiliations:** 1 Cognitive Neurology Center, Saint Louis - Lariboisiere - Fernand Widal Hospital, AP-HP, Université Paris Diderot, Sorbonne Paris Cité, 200 rue du Faubourg Saint Denis, 75010 Paris, France; 2 INSERM UMR1153, Centre of Research in Epidemiology and StatisticS, Paris Descartes University, AP-HP, Sorbonne Paris Cité, Paris, France; 3 Department of biochemistry, Saint Louis - Lariboisiere - Fernand Widal Hospital, AP-HP, Paris, France; 4 Department of Epidemiology and Public Health, University College London, London, UK

**Keywords:** Alzheimer’s disease, CSF biomarkers, β-amyloid peptide, Mortality

## Abstract

**Objective:**

Alzheimer’s disease (AD) is the sixth leading cause of death, with an average survival estimated between 5 and 10 years after diagnosis. Despite recent advances in diagnostic criteria of AD, few studies have used biomarker-based diagnostics to determine the prognostic factors of AD. We investigate predictors of death and institutionalization in a population of AD patients with high probability of AD physiopathology process assessed by positivity of three CSF biomarkers.

**Methods:**

Three hundred twenty-one AD patients with abnormal values for CSF beta-amyloid peptide (Aβ42), tau, and phosphorylated tau levels were recruited from a memory clinic-based registry between 2008 and 2017 (Lariboisiere hospital, Paris, France) and followed during a median period of 3.9 years. We used multivariable Cox models to estimate the hazard ratio (HR) of death and institutionalization for baseline clinical data, genotype of the apolipoprotein E (*APOE*), and levels of CSF biomarkers.

**Results:**

A total of 71 (22%) patients were institutionalized and 57 (18%) died during the follow-up. Greater age, male sex, lower MMSE score, and lower CSF Aβ42 level were associated with an increased risk of mortality. One standard deviation lower CSF Aβ42 (135 pg/mL) was associated with a 89% increased risk of death (95% CI = 1.25–2.86; *p* = 0.002). This association was not modified by age, sex, education, *APOE* ε4, and disease severity. There was no evidence of an association of tau CSF biomarkers with mortality. None of the CSF biomarkers were associated with institutionalization.

**Conclusions:**

Lower CSF Aβ42 is a strong prognostic marker of mortality in AD patients, independently of age or severity of the disease. Whether drugs targeting beta-amyloid peptide could have an effect on mortality of AD patients should be investigated in future clinical trials.

## Background

Patients with Alzheimer’s disease (AD) have a reduced life expectancy compared to the general population, with an average survival estimated between 5 and 10 years after diagnosis [1–3]. AD is currently the sixth leading cause of death [4], and its contribution to mortality is thought to be underestimated due to under-diagnoses of AD [5] and is likely to increase rapidly due to the population aging and the resulting increase in number of persons with AD [6].

The determinants of mortality in AD patients remain unclear; age of the patient and severity of the disease have been shown to be important [2, 7, 8]. An important limitation is the use of clinical criteria for AD diagnosis due to their poor specificity, estimated to be lower than 70% [9]; these criteria do not allow various other causes of cognitive impairment (vascular dementia, Lewy body disease, psychiatric disorders, etc.) to be ruled. The use of tau and β-amyloid peptide (Aβ) biomarkers in diagnostic criteria to reflect the hallmarks of AD neuropathology, and their progressive generalization in clinical setting, allows more accurate diagnosis of AD [10]. These biomarkers have now been incorporated in research diagnostic criteria proposed by the National Institute on Aging and Alzheimer’s Association (NIA-AA) [11], and the International Working Group [12]. More recently, a “biological definition” of AD has been proposed, the A/T/N classification which uses biomarkers of β-amyloid pathology (A), tau lesions (T), and neurodegeneration markers (N) [13, 14].

Despite advances in diagnostic criteria of AD, few studies have used these biomarker-based diagnostics to determine the prognostic factors of AD. Accordingly, the aim of the present study is to investigate the prognostic value of baseline clinical data, ε4 allele of the apolipoprotein E (*APOE*) gene, and CSF biomarkers as determinants of mortality and institutionalization in persons diagnosed with AD. To ensure homogeneity in the population of patients, we restricted the analysis to patients with a clear CSF biomarker profile, i.e., abnormal values for all three CSF biomarkers (Aβ42, total tau, phosphorylated tau (p-tau 181)), corresponding to the recently proposed A+T+N+ profile [13].

## Methods

### Study population

Patients were recruited from a memory clinic-based registry (the BioCogBank study, Lariboisiere hospital, Paris, France) [15]. All patients included in this registry underwent an assessment of CSF AD biomarkers between 2008 and 2017 to explore a cognitive disorder. Subjects were selected for the present study if they were diagnosed with probable AD according to the NIA-AA criteria [11], had data on *APOE* genotype, and if CSF Aβ42 level was below cut-off and CSF tau and p-tau 181 above the cut-off (A+T+N+ profile). Level of education (low: primary school or less, intermediate: secondary to high school, high: baccalaureate or university degree) was recorded, as well as the age at the first consultation. Severity of disease was assessed using the Mini-Mental Status Examination (MMSE) score at the time of lumbar puncture.

### CSF analysis

Lumbar puncture was performed on patients in a fasting state, typically between 9 and 12 in the morning. CSF was collected in 10-mL polypropylene tubes (January 2008 to November 2012: CML model TC10PCS; December 2012 to 2018: Sarstedt catalog no. 62.610.201). CSF samples were centrifuged at 1000*g* for 10 min at 4 °C within 4 h of collection and then aliquoted in 1.5 mL polypropylene tubes and stored at − 80 °C for further analysis (Eppendorf® 0030 120.086 EU). CSF levels of Aβ42, total tau, and p-tau-181 were measured with the commercially available sandwich ELISA INNOTEST®, using the manufacturer’s procedures (Fujirebio Europe NV, formely Innogenetics NV). CSF levels of Aβ40 were only assessed systematically starting in September 2012, using ELISA INNOTEST® from Fujirebio.

Cut-offs were those used in clinical setting, determined as the optimal cut points (e.g., using the Youden index) to differentiate clinically diagnosed AD patients from other disorders and cognitively normal controls [16, 17]. As the type of the polypropylene tube changed in November 2012, cut-offs were adjusted after this date. The cut-offs for CSF Aβ42, tau, and p-tau 181 moved from 500/300/65 pg/mL to 730/300/58 pg/mL after the change in tube is used. The Alzheimer’s Association quality control program for CSF biomarkers validated the quality of CSF evaluations of the hospital biochemistry department [18].

### Outcomes measures

Vital status and institutionalization on June 1, 2018, were determined for each participant. When the patient could not be reached directly, we contacted their relatives or personal physicians to determine their status. Date of institutionalization and/or death was recorded; the cause of death was not available.

### *APOE* genotyping

Venous blood was drawn from participants after informed consent. Genomic DNA was extracted from 200 μL of frozen blood using automated procedures and dedicated DNA purification kits (Maxwell 16, Promega, Madison, WI, USA). After amplification of exon 4 of the *APOE* gene, the *APOE* allelic patterns were identified using denaturing high-performance liquid chromatography (WAVE DNA fragment analysis system, Transgenomic, Omaha, NE, USA) with appropriate controls using the method described previously [19].

### Statistical analyses

Participant characteristics were presented overall, according to their *APOE* ε4 allele status (none ε4, one ε4, two ε4) and their outcome status at the end of the follow-up (death yes/no, institutionalization yes/no). Proportions were calculated for categorical variables, while means and standard deviations were computed for continuous variables. Normality of the distributions of the continuous variables was checked using Shapiro-Wilk test, and CSF Aβ42 was the only variable to be normally distributed. Statistical significance was assessed using a *χ*^2^ test or Student *t* test (CSF Aβ42) or Wilcoxon-Mann-Whitney test (other continuous variables) as appropriate.

Follow-up for mortality and institutionalization was defined as the time between the first consultation at the memory clinic and date of death/institutionalization or the end of follow-up (January 1, 2018), whichever came first. As univariate analyses showed levels of CSF Aβ42 to be lower in patients who died or were institutionalized during the follow-up, we calculated the cumulative incidence of survival and being free of institutionalization as a function of tertiles of CSF Aβ42 using the Kaplan-Meier method and compared them with the log rank test. As the nature of the polypropylene tube has a strong influence on CSF Aβ42 measurement [16], tertiles of CSF Aβ42 were specific for the collection tube used (before and after November 2012). In further analyses, we checked that use of quartiles or quintiles instead of tertiles of CSF Aβ42 led to similar findings. We then used Cox proportional hazard models to estimate hazard ratios for death and institutionalization. The proportional hazards assumptions were checked by computing Schoenfeld residuals. We used tests of interaction to assess whether observed associations were robust across age, sex, education, *APOE* ε4, and disease severity (MMSE). The following parameters were included in the multivariable Cox model: age, sex, MMSE, level of education, *APOE* ε4 status, collection tube for CSF, CSF Aβ42, and CSF tau.

Age, MMSE, and levels of CSF biomarkers were standardized to z-scores (mean = 0, SD = 1) to estimate hazard ratios in order to allow comparisons of the strength of associations of different markers with mortality and institutionalization. The use of z-scores as continuous variables ensured that specific thresholds used to categorize predictors did not drive the results. Stratified analyses were performed to assess the association between CSF Aβ42 and mortality in different subgroups defined by sex, median age, level of education, tertiles of MMSE, and *APOE* ε4 status. Analyses were also stratified by the type of tube used for collection of CSF (before and after November 2012) to ensure that results were not affected by modality of collection of CSF Aβ42. CSF tau and CSF phosphorylated tau were highly correlated in our sample leading us to use CSF tau in the main analyses; use of CSF p-tau 181 instead of CSF tau led to similar findings and conclusions.

All resulting *p* values were two-tailed, and *p* ≤ 0.05 was considered statistically significant. Statistical analyses were performed using SAS version 9.3 (SAS Institute, Cary, NC, USA).

## Results

**Fig. 1:**
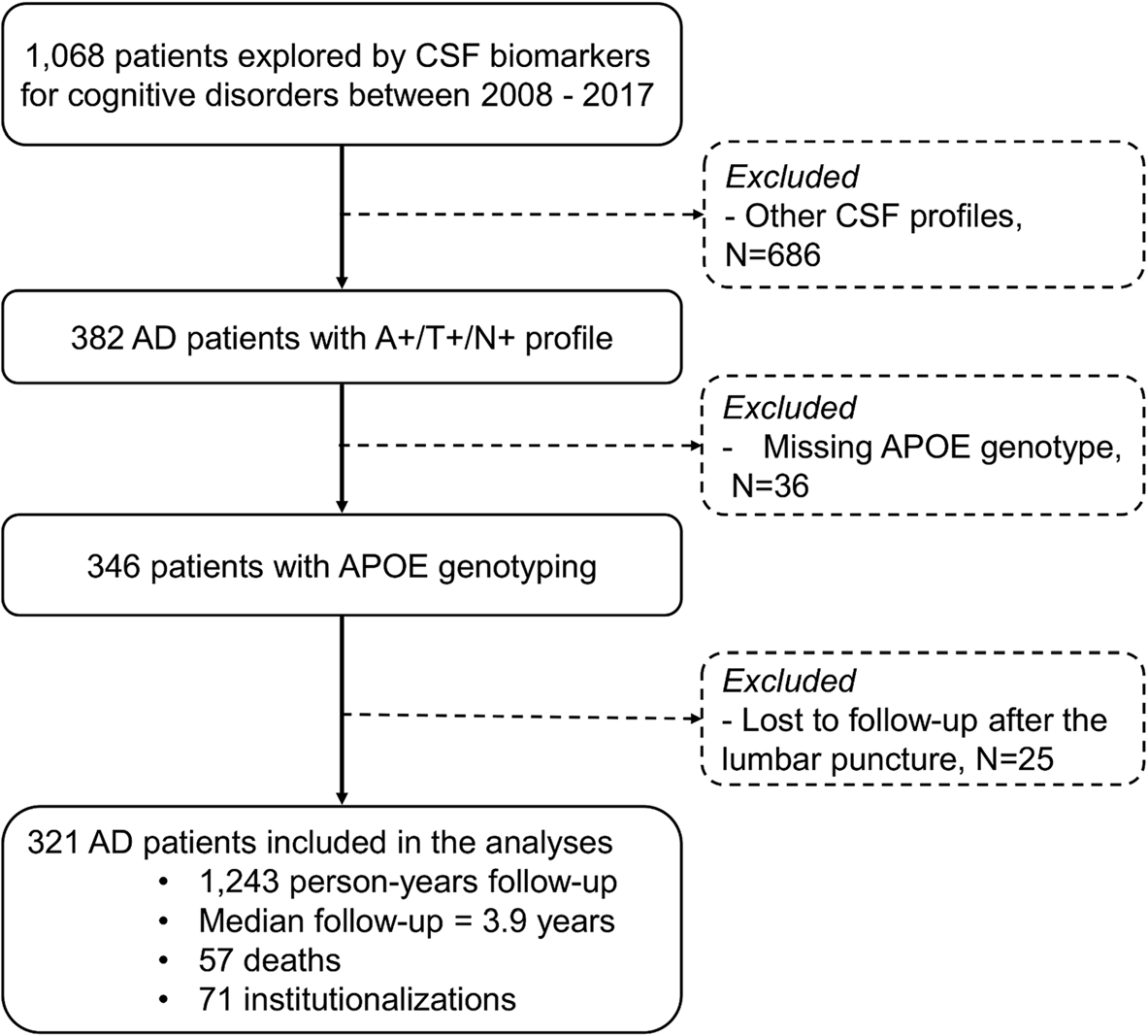
Flow-chart of the study. Selection of the patients for the analyses

Between 2008 and 2017, 1068 patients underwent CSF biomarker assessment for possible cognitive disorders in our memory clinic (flow-chart presented in Fig. 1). Among them, 382 patients (35.8%) had a diagnosis of AD with an A+T+N+ CSF biomarker profile. Thirty-six patients were excluded due to missing data for *APOE* genotype, and 25 patients were lost of follow-up and their mortality/institutionalization status could not be determined. A total of 321 patients were included in the analysis; no differences in age, sex, MMSE, or biomarkers levels were observed between included and non-included patients (Table 1). Subsequent analyses were based on 321 persons, corresponding to a follow-up of 1243 person-years. Among them, 71 patients (22%) were institutionalized and 57 patients (18%) died during a median of follow-up of 3.9 years.

**Table 1:** Comparison between AD patients (A+T+N+ classification) who were included and not included in the analyses due to missing data

Characteristics	AD patients with A+T+N+ profile		
	Included	Not included	
	(*N* = 321)	(*N* = 61)	*P* value
Age, year, mean (SD)	71.0 (8.5)	71.3 (8.1)	0.68
Women, *n* (%)	200 (62.3)	37 (59.7)	0.70
MMSE, mean (SD)	20.3 (5.8)	20.3 (6.7)	0.85
Level of education, *n* (%)^a^			
Low	106 (35.8)	15 (31.9)	
Intermediate	110 (36.8)	11 (23.4)	
High	81 (27.4)	21 (44.7)	0.04
CSF biomarkers, pg/mL, mean (SD)			
Aβ42	483.8 (134.8)	471.8 (133.7)	0.52
Aβ40^b^	13,345 (5447)	13,323 (4812)	0.92
Ratio Aβ42/40^b^	0.047 (0.022)	0.044 (0.017)	0.57
Tau	684.9 (276.9)	671.8 (285.9)	0.72
p-Tau 181	102.6 (39.0)	100.9 (39.5)	0.73

A+T+N+ profile: Abnormal values for three CSF biomarkers (Aβ42, total tau, phosphorylated tau (p-tau 181))

*AD* Alzheimer’s disease, *SD* standard deviation

^a^Low: primary school or less, intermediate: secondary to high school, high: baccalaureate or university degree

^b^CSF Aβ40 was available for 255 patients

Baseline characteristics of the population overall and as a function of the *APOE* ε4 genotype are summarized in Table 2. The mean (SD) age at baseline was 71.0 (8.5) years, 62% were women, 61% were carrying at least one *APOE* ε4 allele, and the mean (SD) MMSE score was 20.3 (5.8). The *APOE* ε4 alleles were associated only with CSF Aβ42 where the mean was lower in AD patients with homozygous ε4/ε4 compared to other patients (Table 2).

**Table 2:** Baseline characteristics of the study population, overall, and stratified by *APOE* ε4 genotype

Characteristics		*APOE* genotype			
	AD patients	0 ε4	1 ε4	2 ε4	
	(*N* = 321)	(*N* = 125)	(*N* = 141)	(*N* = 55)	*P* value
Age, year, mean (SD)	71.0 (8.5)	71.4 (9.9)	71.4 (7.6)	69.0 (6.9)	0.07
Women, *n* (%)	200 (62.3)	76 (60.8)	87 (61.7)	37 (67.3)	0.70
MMSE, mean (SD)	20.3 (5.8)	20.4 (5.6)	19.8 (5.9)	21.4 (5.8)	0.16
Level of education, *n* (%)					
Low	106 (35.7)	48 (40.3)	44 (33.9)	14 (29.2)	
Intermediate	110 (37.0)	38 (31.9)	50 (38.5)	22 (45.8)	
High	81 (27.3)	33 (27.7)	36 (27.7)	12 (25)	0.49
*APOE* ε4 carriers, *n* (%)	196 (61.1)	–	–	–	–
Years of follow-up, mean (SD)	3.9 (2.4)	3.8 (2.5)	3.8 (2.4)	4.1 (2.6)	0.74
Death, *n* (%)	57 (17.8)	25 (20.0)	24 (17.0)	8 (14.6)	0.65
Institution, *n* (%)	71 (22.1)	26 (20.8)	35 (24.8)	10 (18.2)	0.54
CSF biomarkers, pg/mL, mean (SD)					
Aβ42	483.8 (134.8)	481.9 (136.1)	512.1 (126.1)	415.9 (130.8)	< 0.001
Aβ40^b^	13,298 (5454)	12,820 (5648)	13,843 (5498)	12,928 (4897)	0.19
Ratio Aβ42/40^b^	0.047 (0.022)	0.049 (0.022)	0.047 (0.024)	0.040 (0.014)	0.08
Tau	684.9 (276.9)	677.4 (271.4)	705.2 (291.9)	649.5 (248.1)	0.61
p-Tau 181	102.6 (39.0)	103.6 (46.4)	103.8 (35.0)	97.4 (29.6)	0.60

^a^Low: primary school or less, intermediate: secondary to high school, high: baccalaureate or university degree

^b^CSF Aβ40 was available for 214 patients

Patients who died during the follow-up were older, more often men, had lower baseline MMSE scores, and had a lower level of CSF Aβ42 compared to those who survived (Table 3). Patients who were institutionalized had a lower MMSE score and a lower level of CSF Aβ42 compared to those who were not institutionalized. No differences for either outcome were observed for CSF tau or CSF p-tau 181.

**Table 3:** Characteristics associated with death and institutionalization during the follow-up

Characteristics	Death			Institutionalization		
	No	Yes		No	Yes	
	(*N* = 264)	(*N* = 57)	*P* value	(*N* = 250)	(*N* = 71)	*P* value
Age, year, mean (SD)	70.4 (8.2)	73.8 (9.3)	0.001	71.0 (8.2)	70.9 (9.5)	0.89
Women, *n* (%)	172 (65.2)	28 (49.1)	0.02	159 (63.6)	41 (57.8)	0.39
MMSE, mean (SD)	20.7 (5.6)	18.3 (6.6)	0.008	20.7 (5.9)	18.9 (5.5)	0.01
Level of education, *n* (%)						
Low	82 (33.7)	24 (44.4)		86 (37.7)	20 (29.0)	
Intermediate	92 (37.9)	18 (33.3)		77 (33.8)	33 (47.8)	
High	69 (28.4)	12 (22.2)	0.32	65 (28.5)	16 (23.2)	0.10
*APOE* ε4, *n* (%)						
0	100 (37.9)	25 (43.9)		99 (39.6)	26 (36.6)	
1	117 (44.3)	24 (42.1)		106 (42.4)	35 (49.3)	
2	47 (17.8)	8 (14.0)	0.65	45 (18.0)	10 (14.1)	0.54
CSF biomarkers, pg/mL, mean (SD)						
Aβ42	505.9 (127.8)	381.6 (118.7)	< 0.001	496.6 (135.9)	438.7 (121.3)	0.001
Aβ40^b^	13,284 (5362)	13,474 (6697)	0.51	13,195 (5351)	14,165 (6312)	0.36
Ratio Aβ42/40^b^	0.047 (0.022)	0.039 (0.013)	0.08	0.046 (0.019)	0.049 (0.038)	0.56
Tau	680.9 (273.0)	703.6 (296.2)	0.69	685.4 (280.0)	683.2 (267.5)	0.93
p-Tau 181	102.5 (41.0)	102.9 (28.3)	0.88	102.5 (39.5)	102.8 (37.4)	0.94

^a^Low: primary school or less, intermediate: secondary to high school, high: baccalaureate or university degree

^b^CSF Aβ40 was available for 214 patients

**Fig. 2:**
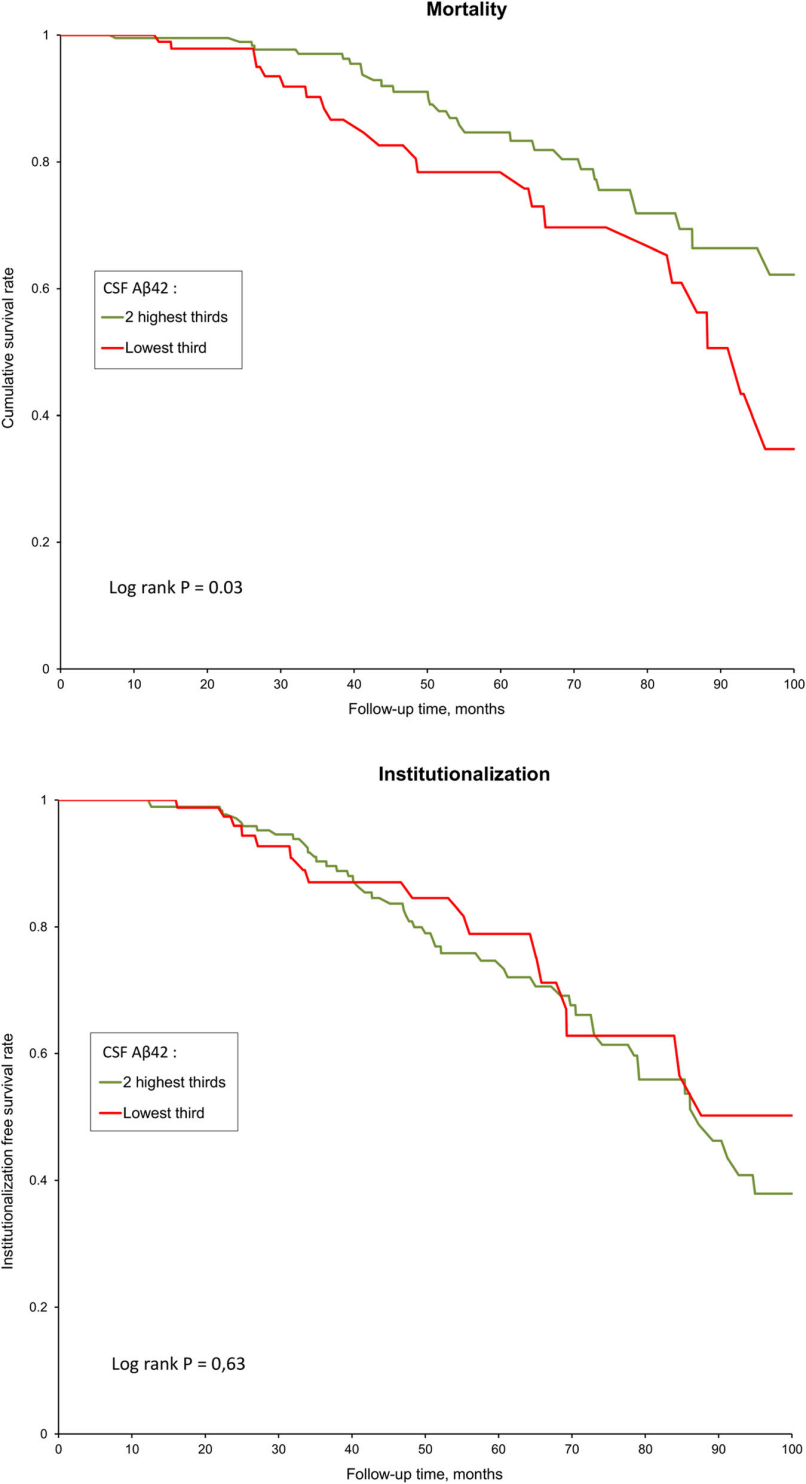
Kaplan-Meier estimates of the cumulative incidences of survival and free of institutionalization, according to lower tertile of CSF Aβ42 compared to the two higher tertiles. Cut-offs used for definition of lower tertile of CSF Aβ42: ≤ 336 ng/mL before November 2012, ≤ 494 ng/mL after December 2012

Figure 2 shows the Kaplan-Meier curves for mortality and institutionalization by tertiles of CSF Aβ42. As no differences were observed for the 2^d^ and 3^d^ tertiles, they were combined and compared to the lowest tertile. Kaplan-Meier curves for mortality began to separate 24 months after baseline assessment and continued to diverge afterwards. After 100 months of follow-up, 35% of the patients in the lower tertile of CSF Aβ42 were still alive, versus 62% for those in the top 2 tertiles (P for log rank = 0.029). When considering lower quartile (*p* = 0.031) or lower quintile (*p* = 0.030) instead of the lowest tertile for CSF Aβ42, the findings were similar. We observed no association between baseline CSF Aβ42 and subsequent institutionalization (Fig. 2).

The multivariable Cox regression analyses for the risk of death/institution are shown in Table 4. Higher age, male sex, lower MMSE score, and lower CSF Aβ42 level were associated with an increased risk of mortality. One standard deviation lower CSF Aβ42 was associated with 89% increased risk of death (95% CI = 1.25–2.86; *p* = 0.002). In this multivariate model, the association of 100 pg/mL lower CSF Aβ42 with mortality was similar to the association observed for 7.9 years greater age or 6.3 points lower score on the MMSE. Men had two times higher risk of death compared to women (HR [95% CI] = 2.11 [1.19–3.76], *p* = 0.01). We observed no association of level of education, *APOE* genotype, or level of CSF tau with mortality. When considering the amyloid ratio (Aβ42/40) in the multivariable model, an association with mortality was also observed. One standard deviation lower amyloid ratio was associated with a twice higher risk of death over the follow-up (HR [95% CI] = 2.10 [1.01–4.39], *p* = 0.04).

**Table 4:** Predictors of death and institution over the follow-up, multivariable Cox regression analyses

Characteristics	Death		Institutionalization	
	HR (95% CI)	*P* value	HR (95% CI)	*P* value
Age, year^a^	1.66 (1.21–2.28)	0.002	1.11 (0.86–1.44)	0.44
Men vs women	2.11 (1.19–3.76)	0.01	1.56 (0.94–2.59)	0.09
Level of education^b^				
Low	Ref.	–	Ref.	–
Intermediate	1.21 (0.58–2.52)	0.61	2.45 (1.25–4.83)	0.009
High	1.02 (0.45–2.31)	0.96	1.45 (0.68–3.09)	0.33
MMSE score^c^	1.56 (1.16–2.10)	0.003	1.68 (1.29–2.18)	< 0.001
*APOE* ε4 allele				
0	Ref.	–	Ref.	–
1	0.79 (0.43–1.44)	0.44	1.00 (0.58–1.73)	0.98
2	0.59 (0.25–1.36)	0.21	0.70 (0.32–1.52)	0.37
Collection tube, before 2012 vs recent	1.37 (0.60–3.01)	0.46	0.74 (0.36–1.52)	0.41
CSF Aβ42, pg/mL^c^	1.89 (1.25–2.86)	0.002	0.89 (0.60–1.32)	0.55
CSF tau, pg/mL^a^	1.11 (0.84–1.48)	0.45	0.99 (0.78–1.26)	0.96

^a^Continuous, estimate for 1 standard deviation increase

^b^Low: primary school or less, intermediate: secondary to high school, high: baccalaureate or university degree

^c^Continuous, estimate for 1 standard deviation decrease

The predictors had a less robust association with risk of institutionalization; intermediate level of education and lower baseline MMSE score were associated with increased risk of institutionalization. Men tended to be at higher risk than women (HR [95% CI] = 1.56 [0.94–2.59), *p* = 0.09). We found no effect of *APOE* genotype or CSF biomarkers on the risk of institutionalization.

**Fig. 3:**
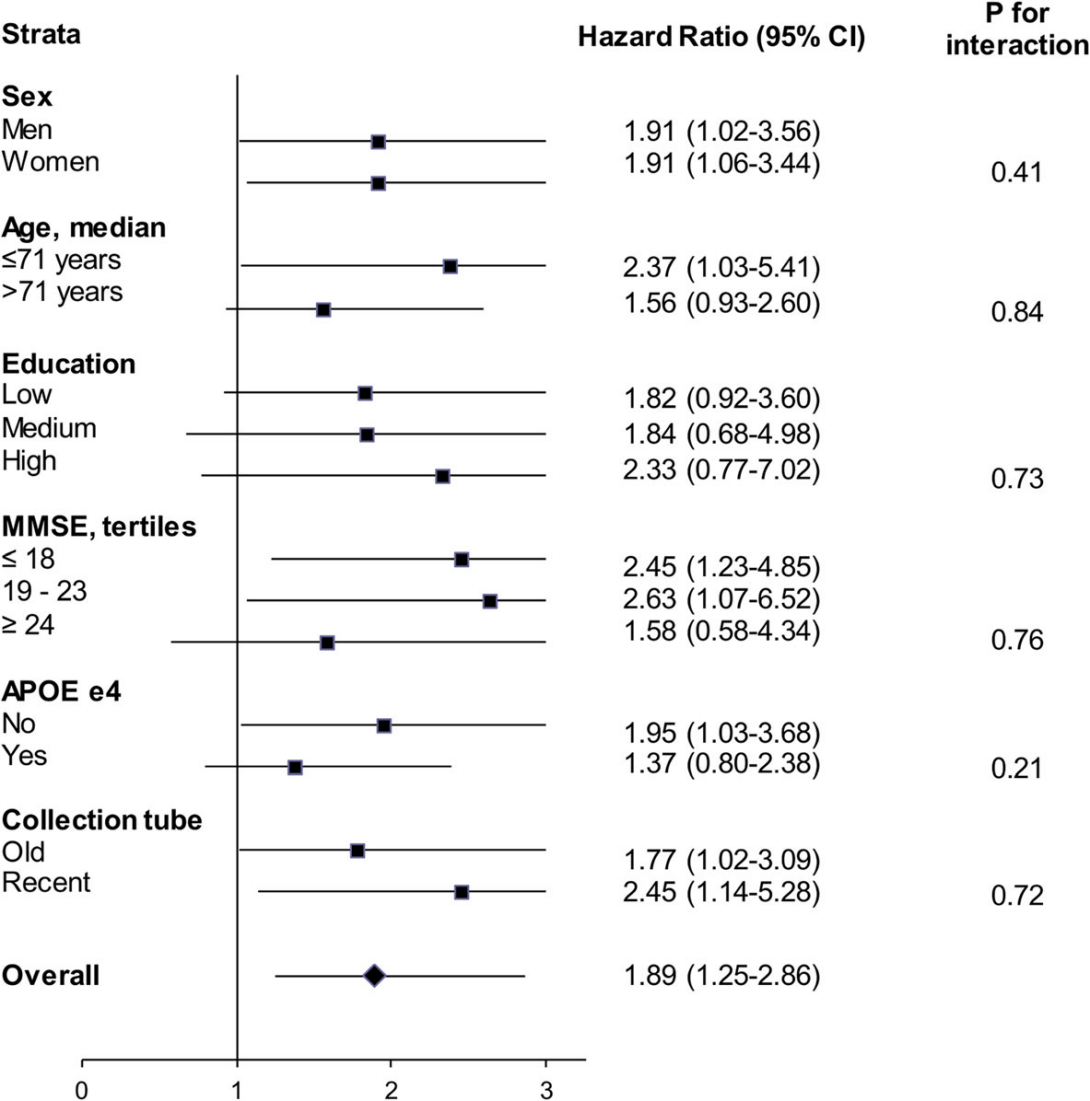
CSF Aβ42 and risk of death over follow-up: stratified analyses. Hazard ratios estimate relative risk of death for a decrease of one standard deviation of CSF Aβ42, considered as a continuous variable. Models are adjusted for age, sex, education level, APOE genotype, type of collection tube, MMSE, and CSF tau

Figure 3 presents the results of stratified analyses for the risk of death as a function of one standard deviation decrease in CSF Aβ42; there was no robust evidence of difference in associations in the subgroups. Reassuringly, the type of tube used to collect CSF data (before and after November 2012) did not influence the association between CSF Aβ42 level and mortality.

## Discussion

In this study, including 321 AD patients, with verification of AD physiopathology using positivity of the three widely used CSF biomarkers, we found that lower levels of CSF Aβ42 to be associated with an increased risk of death. This association was clinically significant as the effect of 100 ng/mL lower CSF Aβ42 on mortality was similar to that of 8 years of aging. We show the main effects of previously known risk factors such as age, male sex, and severity of the disease to be similar to those in other studies, providing evidence of the robustness of our results. There was also evidence of the importance of the amyloid ratio (Aβ42/40) for mortality. We found no association between *APOE* genotype and tau biomarkers on mortality outcomes in AD patients. The risk of institutionalization in AD patients was associated with poorer MMSE scores, used here as a proxy for severity of AD, but CSF biomarkers, *APOE* genotype, and age were not associated with risk of institutionalization.

Few previous studies have examined whether AD biomarkers are associated with prognostic outcomes such as mortality and institutionalization, and to the best of our knowledge, none have used biomarker-based criteria for the ascertainment of AD status in order to ensure homogeneity in the population of patients. A previous study, based on 127 patients diagnosed with mild cognitive impairment or dementia, found an association between amyloid deposition assessed by amyloid PET imaging and mortality; however, 37% of those included in the analysis were negative for the amyloid marker [20]. Another paper based on 196 AD patients reported an association between CSF tau and mortality [20]. Here again, 46% of these patients had at least one of the three CSF biomarkers in the normal range. More recently, a study on 616 AD patients, diagnosed according to clinical and not biological criteria, failed to find an association between CSF biomarkers and mortality [21].

Despite the association of *APOE* genotype with life expectancy [22], its association with mortality in AD patients remains unclear [23]. We found no significant effect of *APOE* genotype on mortality, a finding that is consistent with those of several other previous studies [21, 24]. Homozygous ε4/ε4 patients tended even to have a lower risk of dying during follow-up, which may be partly explained by their younger age.

Several studies have shown an inverse association between CSF Aβ42 and total brain amyloid load, assessed by neuropathology [25, 26] or by amyloid PET imaging [27, 28]. The association between lower CSF Aβ42, which reflects higher level of amyloid deposits in the brain, and increased mortality may therefore be explained by more advanced disease which contributes to risk of death. However, the association we found was not substantially attenuated by the adjustment for severity of AD as assessed by the MMSE score, and we found no effect of CSF Aβ42 on the risk of institutionalization. There is increasing evidence of shared molecular mechanisms between AD and atherosclerosis [29, 30], which would point towards the role of Aβ peptide in vascular inflammation pathophysiology [31, 32]. Plasmatic dosage of beta-amyloid peptide has also shown to be associated with global [33] and cardiovascular [34] mortality. It is possible that patients with more pronounced brain amyloid pathology have a higher risk of cardiovascular mortality. Several drugs targeting Aβ peptide are currently being investigated in on-going trials [35]. Beyond their hoped effect on the cognition of patients, our results suggest also a hypothetical positive effect on survival in AD patients. Interestingly, in the negative solanezumab trial, the rate of cardiac disorders at 18 months tended to be lower in the treated group compared to the placebo group (1.6% vs 2.9%, *p* = 0.06), this was also the case for cerebrovascular accidents (0.0% vs 0.5%, *p* = 0.06) [36].

This study has several strengths, including its large size, a low rate of attrition over the follow-up, exclusive focus on patients positive for all three CSF biomarkers, and the fact that the analyses of CSF were performed in a single biochemistry department which ensures that inter-site variability does not affect results [16]. One of the limitations is the lack of data on the exact cause of death which precluded more detailed analyses on specific causes of mortality. Another limitation is the variability in the measurement of CSF Aβ42 which is likely to result in dilution of the association. The recent development of automated platforms for dosing AD biomarkers may in the future contribute to decrease this variability.

## Conclusions

In summary, we found in biomarker-positive AD patients that CSF Aβ42 rather than tau biomarkers is associated with the risk of subsequent mortality. Whether drugs targeting beta-amyloid peptide could have also an effect on mortality of patients may deserve to be investigated as an outcome in future clinical trials.

## Abbreviations

AD: Alzheimer’s disease
A/T/N: β-Amyloid (A), tau lesion (T), and neurodegeneration (N) classification
Aβ42: β-Amyloid peptide
CI: confidence interval
HR: Hazard ratio
MMSE: Mini-Mental Status Examination
p-Tau 181: Phosphorylated tau
